# Hearing function after betahistine therapy in patients with Ménière's disease^☆^

**DOI:** 10.1016/j.bjorl.2015.08.021

**Published:** 2015-12-18

**Authors:** Seyed Javad Seyed Tootoonchi, Samad Ghiasi, Parvaneh Shadara, Simin Mirakhor Samani, Daniel Fadaei Fouladi

**Affiliations:** a Tabriz University of Medical Sciences, Imam Reza Teaching Center, Department of ENT, Tabriz, Iran; b Tabriz University of Medical Sciences, Drug Applied Research Center, Tabriz, Iran; c Tabriz University of Medical Sciences, Neurosciences Research Center, Tabriz, Iran

**Keywords:** Medical treatment, Ménière's disease, Hearing loss, Prognosis, Tratamento médico, Doença de Ménière, Perda de audição, Prognóstico

## Abstract

**Introduction:**

Preventing or reversing hearing loss is challenging in Ménière's disease. Betahistine, as a histamine agonist, has been tried in controlling vertigo in patients with Ménière's disease, but its effectiveness on hearing problems is not known.

**Objective:**

To examine the effect of betahistine on hearing function in not-previously-treated patients with Ménière's disease and to define possible contributors in this regard.

**Methods:**

A total of 200 not-previously-treated patients with definite unilateral Ménière's disease received betahistine by mouth (initial dose, 16 mg three times a day; maintenance dose, 24–48 mg daily in divided doses). Changes in indicators of hearing status before and six months after treatment were documented. Hearing loss was considered as the mean hearing level >25 dB HL at five frequencies.

**Results:**

The mean duration of disease was 3.37 years. Six months after treatment the mean hearing level decreased by 6.35 dB compared to that at the baseline (*p* < 0.001). Both patients’ age and the duration of disease correlated negatively with the improvement in hearing function. Post treatment hearing loss was independently associated with age, the initial hearing level and the chronicity of disease. The corresponding optimal cut-off points for predicating a persistent hearing loss 6 months after treatment were 47 years, 38 dB HL, and 1.4 years, respectively.

**Conclusion:**

Oral betahistine was significantly effective in preventing/reversing hearing deterioration in patients with Ménière's disease. Age, the hearing level on admission, and the disease duration were independent predictors of hearing status after treatment.

## Resumo

**Introdução:**

Prevenir ou reverter a perda auditiva é um desafio na doença de Ménière. A beta-histina, um agonista de histamina, tem sido testada no controle de vertigem em pacientes com doença de Ménière, mas sua eficácia em problemas de audição ainda não é conhecida.

**Objetivo:**

Analisar o efeito da beta-histina na função auditiva em pacientes com doença de Ménière não tratados previamente, e definir possíveis contribuintes a esse respeito.

**Método:**

Um total de 200 pacientes sem tratamento prévio, e com diagnóstico definido de doença de Ménière unilateral, recebeu beta-histina por via oral (dose inicial de 16 mg três vezes ao dia; dose de manutenção de 24–48 mg por dia, em doses divididas). Alterações do limiar auditivo antes e após seis meses de tratamento foram documentadas. Considerou-se como perda auditiva uma média do nível de audição >25 dB NA em cinco frequências.

**Resultados:**

A média de duração da doença foi de 3,37 anos. Seis meses após o tratamento, a média do limiar auditivo diminuiu em 6,35 dB, em comparação com o valor da linha de base (p < 0,001). Tanto a idade dos pacientes quanto a duração da doença apresentaram correlação negativa com a melhora da função auditiva. A perda auditiva após o tratamento foi independentemente associada à idade, ao nível inicial de audição e à cronicidade da doença. Os pontos de corte ótimos correspondentes para prever uma perda auditiva persistente seis meses após o tratamento foram 47 anos, 38 dB HL e 1,4 ano, respectivamente.

**Conclusão:**

A beta-histina oral foi significantemente eficaz na prevenção/reversão da deterioração auditiva em pacientes com doença de Ménière. Idade, nível de audição na admissão e duração da doença foram fatores preditivos independentes da condição auditiva após o tratamento.

## Introduction

Described for the first time in 1861 by Prosper Ménière, Ménière's disease or idiopathic endolymphatic hydrops is a disorder of the inner ear. Elevated pressure in the endolymph can cause four symptoms: (1) fluctuating sensorineural hearing loss, (2) occasional episodic vertigo, (3) tinnitus, and (4) aural fullness.^1^

The audiometric pattern of hearing loss in Ménière's disease fluctuates by time. While the hearing loss is limited to low frequencies in the early stages, medium and high frequencies are also involved during the progression of disease. Appropriate treatments, however, may prevent, or at least defer hearing loss in Ménière's disease, particularly when they are started early after the onset of symptoms.^2^

Since the disease is a chronic condition that evolves over a rather long period of time, time series analyses are required for evaluating the effectiveness of treatment and the estimation of prognosis.^3^

Betahistine (Serc) is a histamine agonist that has been found effective in symptomatic treatment of Ménière's disease. Although the exact mechanism(s) of action is unknown, betahistine is thought to act by increasing blood flow to the cochlear stria vascularis and/or preventing the activity of the vestibular nuclei.^4^, ^5^

By now, some studies have tried to test the actual usefulness of this medication in Ménière's disease, but because the majority of them are methodologically flawed, reaching a definite conclusion has been impossible. This is why betahistine has not been approved by the US Food and Drug Administration (FDA) to be used in patients with Ménière's disease.^6^

In spite of this, Smith et al.^7^ reported that 94% of otolaryngologists in the United Kingdom prescribe betahistine to their Ménière's patients. Similar reports are available from South American countries.^8^ To the best of the authors’ knowledge, however, the effectiveness of betahistine in preventing or ameliorating hearing loss in patients with Ménière's disease has not been tested. Thus, this study seeks to examine the effect of betahistine therapy on hearing function in patients with no previous treatment for Ménière's disease, and to define possible related contributors.

## Methods

### Patients

A total of 200 not-previously-treated patients with definite Ménière's disease were consecutively recruited from a teaching clinic between 2011 and 2013. The ethics committee of a local university approved this study (no. 5/4/6629), and written informed consents were obtained from the participants.

Subjects with probable/possible Ménière's disease, bilateral involvement, prior otological surgery, or underlying systemic diseases were not included.

The diagnosis and grading of Ménière's disease was according to the guidelines offered by the American Academy of Otolaryngology – Head and Neck Surgery.^9^

Each patient underwent otoscopy and audiometry under quiescent state without vertiginous attack at baseline and six months post treatment.

### Audiometry

A standard audiometer (Amplivox 270, Amplivox, Oxfordshire, England) was used for the measurement of hearing thresholds. At four frequencies (500, 1000, 2000, and 3000 Hz) the four tone average was calculated from the worst audiogram during the interval of three months before and six months after treatment.^9^ The average hearing level at five frequencies (250, 500, 1000, 2000, and 4000 Hz) was used in defining hearing loss (the mean hearing level >25 dB HL).^10^

### Treatment

Patients received betahistine dihydrochloride (Betaserc, 8 mg tablet, Abbott, Illinois, USA) by mouth, initially 16 mg three times a day, with food, at a maintenance dose of 24–48 mg daily divided in doses to control symptoms.^11^, ^12^

General dietary recommendations such as low sodium intake were implemented, but no other medication except for betahistine was allowed during the study period.

### Outcome measures

Improvement in the hearing level six months after treatment (IHL_6_): hearing level at baseline – hearing level 6 months after treatment.

Hearing improvement: IHL_6_ > 0
Hearing loss: six-month post treatment mean hearing level >25 dB HL.^10^

### Statistical analysis

The SPSS software version 19.0 (IBM Corporation, New York, USA) was used for statistical analysis. Distribution of numeric data was tested using the Kolmogorov–Smirnov method. The numeric data were shown as the mean ± standard deviation or the mean [standard error of the mean, SEM]. The contingency tables (Pearson chi-square test), McNemar test, independent samples *t* test, paired samples *t* test, independent Mann–Whitney *U* test, and Wilcoxon signed ranks test were used, when appropriate. The Spearman's coefficient (rho)/Pearson coefficient (*r*) was calculated to investigate correlations between variables. A stepwise descending logistic regression analysis/linear regression model was used in constructing a multivariate analysis. The receiver operator characteristics (ROC) curve was drawn in determining areas under the curve and optimal cut-off values. A significance level was set at *p* ≤ 0.05.

## Results

The study variables including demographic information and general data at the time of admission are summarized in Table 1.

**Table 1:** Demographic data and baseline variables in 200 patients with Ménière's disease.

*Sex*	
Male	67 (33.5)
Female	133 (66.5)

*Age (year)*	45.29 ± 12.16 (18–78)

*Disease duration (month)*	40.44 [1.20] (1–260.4)

*Symptoms*	
Vertigo	200 (100)
Tinnitus/aural fullness	200 (100)
Hearing problems	186 (93)

*Disease stage*	
I	72 (36)
II	71 (35.5)
III	54 (27)
IV	3 (1.5)

*Hearing level (dB HL)*	33.32 [1.27] (10–100)

*Hearing loss*	129 (64.5)

Data are presented as frequency (%), mean ± standard deviation (range), or mean [standard error of the mean] (range).

**Figure 1:**
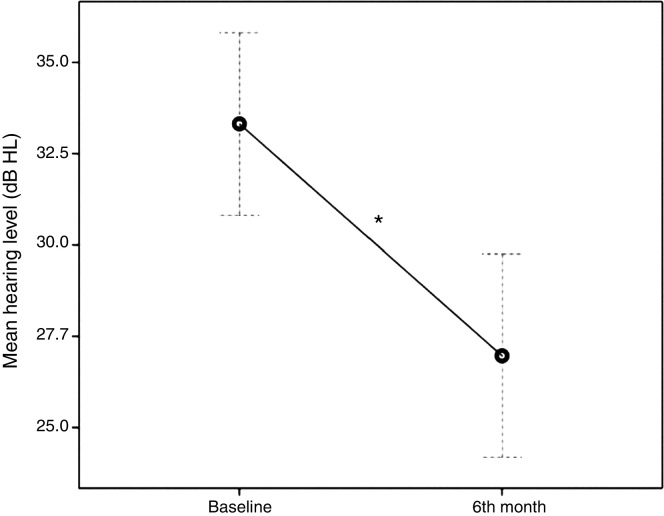
Changes in the mean hearing levels at baseline and six months after treatment. Error bars represent 95% confidence interval. *p ≤ 0.05 is statistically significant.

Six months post treatment the mean hearing level dropped significantly at 26.97 dB HL (SEM, 1.42; percent decrease, 19.06%; Wilcoxon signed ranks test *p* < 0.001) (Fig. 1).

The mean IHL_6_ was 6.35 dB (SEM, 0.75), denoting a hearing improvement in 60 patients (30%) at the endpoint.

Six months after treatment a retained hearing loss was detected in 90 patients, indicating a 30.23% decrease by the treatment (McNemar test *p* < 0.001).

## Contributors to hearing function after treatment

### IHL_6_

**Figure 2:**
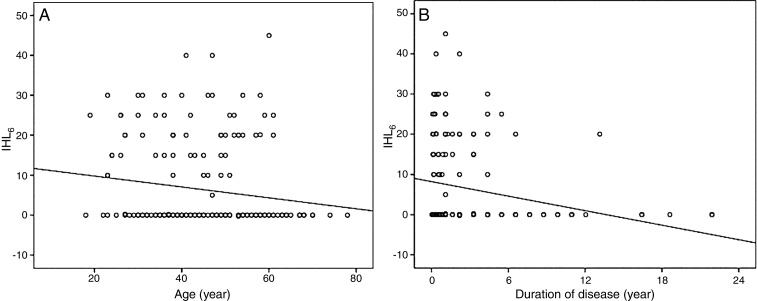
Simple scatterplots representing reverse correlations between patients’ age and improvement in the hearing level six months after treatment (IHL_6_) (A) and between disease duration and IHL_6_ (B).

The mean IHL_6_ was 6.99 dB (SEM, 0.08) in females and 5.07 dB (SEM, 0.14) in males (Mann–Whitney *U* test *p* = 0.23). A significant, reverse correlation was seen between age and IHL_6_ (Pearson *r* = −0.16, *p* = 0.03) (Fig. 2 A), indicating a negative connection between increasing age and improvement in hearing function after treatment. There was no significant correlation between IHL_6_ and the baseline hearing level (Spearman's rho = 0.09, *p* = 0.21), whereas a negative, significant correlation was present between IHL_6_ and the duration of disease (Spearman's rho = −0.31, *p* < 0.001) (Fig. 2B).

In a linear regression model, both age and the duration of disease were independently associated with IHL_6_ (*p* = 0.05 and 0.002, respectively).

### Hearing improvement

Males and females had similar hearing improvement rates after treatment (25.4% vs. 32.3%, respectively; chi-square test *p* = 0.31). Patients with hearing improvement, however, were significantly younger (41.60 ± 11.46 years vs. 46.86 ± 12.14 years; independent samples *t* test *p* = 0.01). The mean disease duration was significantly lower in the cases with hearing improvement than in those with no change or deterioration in hearing function six months after treatment (1.71[0.28] years vs. 3.26[0.39] years; Mann–Whitney *U* test *p* < 0.001). The mean baseline hearing level was 33.75 dB HL (SEM, 1.31) in patients with hearing improvement and 33.14 dB HL (SEM, 1.72) in the remaining ones (Mann–Whitney *U* test *p* = 0.79).

**Figure 3:**
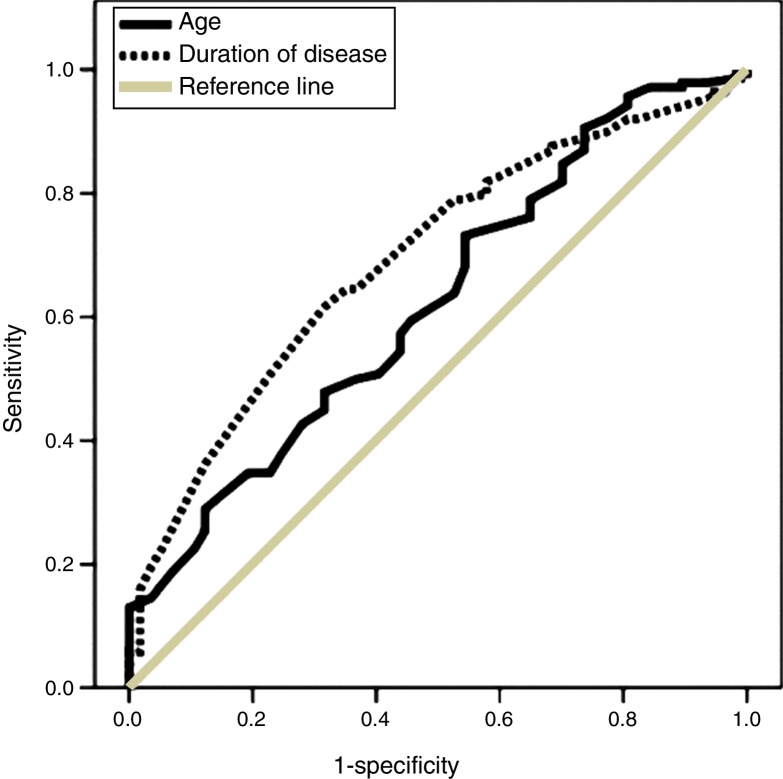
Receiver operator characteristics’ curves of patients’ age and disease duration in predicting unfavorable hearing function six months after treatment. Area under the curve: 0.62 for age and 0.69 for disease duration (*p* = 0.01 and <0.001, respectively).

In multivariate analysis both age and disease duration were independent determinants of post treatment hearing improvement (*p* = 0.01, Exp(*B*) = 0.96 and *p* = 0.002, Exp(*B*) = 1.00, respectively). With areas under the ROC curves of 0.62 (*p* = 0.01) and 0.69 (*p* < 0.001), the related optimal cut-off points were 44 (sensitivity: 57.3%, specificity: 56.1%) and 1.4 (sensitivity: 63.5%, specificity: 68%), respectively (Fig. 3).

### Persistent hearing loss

Variables in relation to persistent hearing loss after treatment are summarized in Table 2. Accordingly, advanced age (independent samples *t* test *p* = 0.01), male gender (chi-square test *p* = 0.05), a more chronic disease (Mann–Whitney *U* test *p* < 0.001) and a high baseline hearing level (Mann–Whitney *U* test *p* < 0.001) were significantly associated with persistent hearing loss after treatment.

**Table 2:** Study variables in patients with persistent and resolved hearing loss (HL) six months after treatment.

Variables	Persistent HL (*n* = 90)	Resolved HL (*n* = 39)	*p*-Value
Sex (female)	53 (58.9)	30 (76.9)	0.05^a^
Age (year)	49.73 ± 12.74	43.15 ± 11.19	0.01^a^
Disease duration (year)	4.28 [0.05]	1.71 [0.06]	<0.001^a^
Hearing level (dB HL)	47.20 [1.45]	35.64 [1.07]	<0.001^a^

Data are shown as frequency (%), mean ± standard deviation, or mean [standard error of the mean].

Hearing loss (HL), indicates mean hearing level of five frequencies >25 dBn HL.

a *p*-Value ≤ 0.05 is statistically significant.

In multivariate analysis, except for sex (*p* = 0.11, Exp(*B*) = 2.41), all the remaining variables including age (*p* = 0.01, Exp(*B*) = 1.06), disease chronicity (*p* = 0.01, Exp(*B*) = 1.00) and baseline hearing level (*p* < 0.001, Exp(*B*) = 1.10) were found as independent contributors to the post treatment hearing function.

**Figure 4:**
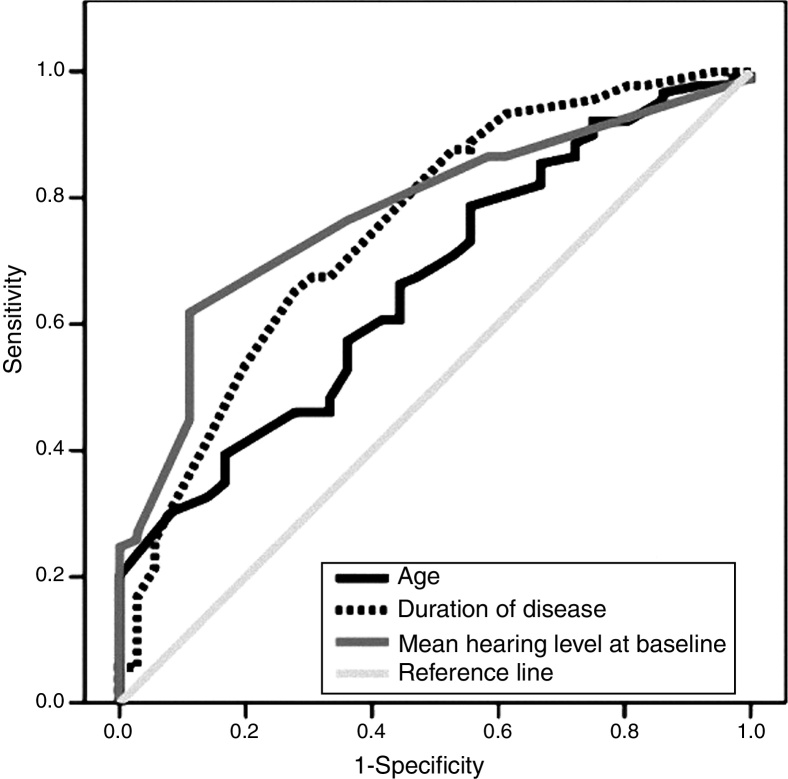
Receiver operator characteristics’ curves of patients’ age, baseline hearing level, and disease duration in predicting persistent hearing loss six months after treatment. Area under the curve: 0.66 for age, 0.76 for baseline hearing level, and 0.75 for disease duration (*p* = 0.01, <0.001, and <0.001, respectively).

The related ROC curves of age, disease chronicity and baseline hearing level are shown in Fig. 4. With areas under the ROC curves of 0.66 (*p* = 0.01), 0.75 (*p* < 0.001) and 0.76 (*p* < 0.001), the optimal cut-off points were 47 (sensitivity: 60.7%, specificity: 58.3%), 1.4 (sensitivity: 67.4%, specificity: 69.4%) and 38 (sensitivity: 76.4%, specificity: 63.9%), respectively.

## Discussion

Although a large variability exists as to the symptoms appearing in the course of Ménière's disease, hearing loss is usually accounted as an inevitable consequence.^13^ In contrast to the outdated notion of “natural history” that suggests neither surgical nor pharmacologic interventions could alter hearing deterioration in Ménière's disease,^14^, ^15^, ^16^ timely diagnosis and early treatment could remarkably impede its progression.^17^ An average improvement of 6.35 dB in hearing level six months after the administration of betahistine in the present study supports this fact.

A younger age and shorter duration of the disease were two independent predictors of post treatment hearing improvement in this work. Similarly, in a study on pure tone audiograms of 300 patients with Ménière's disease, Paparella et al.^18^ found the duration of disease as an important prognostic factor in relation to alterations in hearing function. In another retrospective series, Katsarkas^19^ examined 475 patients suffering from Ménière's disease and found that despite case-to-case variations in hearing loss, the hearing impairment correlated significantly over time with the elapsed time since the onset of the disease. In a retrospective study on 243 patients with Ménière's disease by Havia et al.,^20^ prolonged disease was associated with deterioration of hearing function. In a very recent study by Sato et al.,^21^ potential factors in connection with the prognosis of hearing loss were examined retrospectively in a group of 36 patients with unilateral Ménière's disease. They reported that the mean interval from the onset of the disease to the initial visit was significantly longer in patients with poor hearing. Likewise, a similar connection has been suggested between age and hearing impairment in patients with Ménière's disease.^22^, ^23^ To the best of the authors’ knowledge, however, this is the first study that reports an independent role of age in determining hearing function and its responsiveness to medical treatment with betahistine in Ménière's disease.

Considering the effect of treatment on hearing loss (i.e. the mean hearing level of five frequencies >25 dB HL), the percentage of patients affected by this problem decreased significantly from 64.5% at the baseline to 45% at the endpoint (*p* < 0.001). In addition to age and the duration of disease, admitting hearing level (in multivariate study) and sex (in univariate study) were other predicting factors in this regard.

In terms of hearing prognosis, a compromised hearing ability at the initial visit may augur ill for the future in patients with Ménière's disease.^21^ Sato et al.^21^ also found that the hearing levels at the initial visits were significantly worse in patients with poor than in those with good prognoses.

Cochlear and eighth cranial nerve injuries have been suggested as plausible underlying causes of Ménière's disease by some investigators. Relative preservation of hair cells at the same points of injury further corroborates the theory of neurotoxicity in such patients. It has been found that nerve deterioration is possibly independent of hydrops severity.^24^ This may explain the heterogeneity found in this study in terms of the prognostic role of initial hearing level, which was significantly associated with persistent hearing loss but not with the degree of improvement in hearing function after treatment. That is to say, structural rather than functional abnormalities go along with permanent hearing loss that does not respond adequately to medical treatments. Presence of hearing loss may indicate such structural changes in patients with Ménière's disease.^18^

In a similar study that attempted to identify factors in association with the responsiveness to medical management of Ménière's disease, Devaiah and Ato^25^ studied 29 patients. They found that aggressive medical therapy (sodium restriction and diuretic treatment in this series) may prevent disease progression in terms of hearing loss in those with less severe disease.

In the current work the authors tried to define cut-off points for age, the initial hearing loss, and the duration of disease. Using ROC curve analysis, the corresponding values were 47 (sensitivity: 60.7%, specificity: 58.3%), 38 (sensitivity: 76.4%, specificity: 63.9%), and 1.4 (sensitivity: 67.4%, specificity: 69.4%), respectively.

In a study by Kotimaki et al.^23^ the records of 205 patients with definite diagnosis of Ménière's disease were retrospectively reviewed. They showed, in line with our findings, that both age and the duration of disease are associated with hearing impairment, with deterioration in pure-tone average over the frequencies 0.5–4 kHz by approximately 1 dB per year due to the duration of disease and by approximately 0.5 dB per year because of aging. In this study the age of 50 years was reported as a determinant of the role of the duration of disease in affecting hearing loss. Although the age of 47 years in the present study was associated with persistent hearing loss after treatment independent of the duration of disease, their reported figure approximates ours.

In normal people the rate of hearing loss by aging at various frequencies may differ between males and females. This gender-related difference may explain our finding in terms of the effect of sex on the responsiveness of hearing loss to medical treatment. More studies, however, are needed to draw a solid conclusion in this regard.^23^

In the present work we excluded patients with bilateral disease because bilaterality has been suggested as a predicting factor for poor hearing prognosis in patients with Ménière's disease^1^, ^18^ and it could have interfered with the main objective of this study.

Another limitation of the present work was a relatively short follow-up of hearing condition after treatment (six months). Longer follow-ups may be required in this regard.^26^

Patients recruited in this study were diagnosed recently as cases with Ménière's disease. Medical records and patients’ declarations were the only documents we relied on to evaluate history and possible previous treatments. This may decrease the reliability of employed data in this work.

Despite statistically significant hearing improvements detected in the current study, these changes might not be clinically advantageous. It should be acknowledged that it was not possible to verify if the patients were in an inter-crises period, when the hearing is usually better. That could have happened for the patients presenting with mild losses, and so, reaching uniform clinical outcomes could have been affected adversely.

The final limitation was that only the results of a conservative, not consensually accepted medical treatment (i.e. betahistine) were considered in this study. This approach was chosen because nonpharmacologic treatments are diverse and their outcome varies significantly between studies.^14^, ^15^, ^16^ In addition, as mentioned before, in many countries such as in the United Kingdom and many South American countries this medication is widely used for symptomatic treatment of Ménière's disease.^7^, ^8^

## Conclusion

Based on the findings of this study, oral betahistine is effective in preventing/correcting hearing problems in patients with Ménière's disease. Age and the duration of disease independently contributed to the effectiveness of the medication. Sex and the initial hearing level may also play a role in this regard.

## Conflicts of interest

The authors declare no conflicts of interest.
